# Helicobacter pylori infection as a risk factor for diabetes: a meta-analysis of case-control studies

**DOI:** 10.1186/s12876-020-01223-0

**Published:** 2020-03-24

**Authors:** Kamyar Mansori, Yousef Moradi, Sara Naderpour, Roya Rashti, Ali Baradaran Moghaddam, Lotfolah Saed, Hedyeh Mohammadi

**Affiliations:** 1 Department of Biostatistics and Epidemiology, School of Medicine, Zanjan University of Medical Sciences, Zanjan, Iran; 2 Department of Epidemiology, School of Public Health, Iran University of Medical Sciences, Tehran, Iran; 3 Social Determinants of Health Research Center, Research Institute for Health Development, Kurdistan University of Medical Sciences, Sanandaj, Iran; 4 Research Center of Pediatric Infection Diseases, Institute of Immunology and Infection Diseases, Iran University of Medical Sciences, Tehran, Iran; 5 Department of Endocrinology, Faculty of Medicine, Kurdistan University of Medical Science, Sanandaj, Iran; 6 Faculty of Medicine, Kurdistan University of Medical Science, Sanandaj, Iran

**Keywords:** Helicobacter pylori, Diabetes mellitus, Type 1 diabetes, Type 2 diabetes, Meta-analysis

## Abstract

**Background:**

There are several studies with varied and mixed results about the possible relationship between *H. pylori* and diabetes. Therefore, this current meta-analysis performed to determine the association between *H. pylori* infection and the risk of diabetes mellitus.

**Methods:**

A systematic literature searches of international databases, including Medline (PubMed), Web of Sciences, Scopus, EMBASE, and CINHAL (January 1990–March 2019) was conducted to identify studies investigating the relationship between *H. pylori* infection and diabetes mellitus. Only case–control studies were analyzed using odds ratio (OR) with 95% confidence intervals (CIs). Stratified and subgroup analyses were performed to explore heterogeneity between studies and assess effects of study quality. Logarithm and standard error logarithm odds ratio (OR) were also used for meta-analysis**.**

**Results:**

A total of 41 studies involving 9559 individuals (case; 4327 and control; 5232) were analyzed. The pooled estimate of the association between *H. pylori* infection with diabetes was OR = 1.27 (95% CI 1.11 to 1.45, *P* = 0.0001, I^2^ = 86.6%). The effect of *H. pylori* infection on diabetes mellitus (both types), type 1 and type 2 diabetes was 1.17 (95% CI 0.94 to 1.45), 1.19 (95% CI 0.98 to 1.45), and 1.43 (95% CI 1.11 to 1.85) respectively. Subgroup analysis by the geographical regions showed in Asian population risk of the effect of *H. pylori* infection on diabetes was slightly higher than other population,

**Conclusion:**

In overall a positive association between *H. pylori* infection and diabetes mellitus was found.

## Background

Helicobacter pylori (*H. pylori*) is a gram-negative spiral bacterium which is found abundantly in the stomach. The *H. pylori* infection is one of the most common chronic infections in the world, so that more than 50% of the world’s population are infected with this infection [1, 2]. It is now known that *H. pylori* is responsible for most cases of peptic ulcer disease. Also, the different studies highlighted that it is associated with other important gastrointestinal diseases such as chronic gastritis, gastric adenocarcinoma, and MALT lymphoma which are recognized as a major public health concern in the world [3, 4]. In addition to the role of *H. pylori* in gastrointestinal disorders, some researches have suggested the potential role of this bacterium in the development of non-gastrointestinal disorders such as cardiovascular diseases and metabolic syndrome especially diabetes [5–7]. Diabetes is the most common metabolic disease in the world and responsible for about 4 million deaths per year. The global prevalence of diabetes was 4.6% equivalent to 285 million in adults for 2010, which this number has reached 371 million in 2012, and is expected to reach 552 million by 2030 [8–10].

As mentioned above, one of the factors that may affect incidence of diabetes is *H. pylori*. The relationship between *H. pylori* infection and diabetes was introduced in 1989 [11]. It has been suggested that the *H. pylori* may be contributed to the incidence of cardiovascular disease and diabetes through elevations in inflammatory cytokines levels such as C-reactive protein (CRP) and interleukin-6 [11–13]. In general, various studies have investigated the role of *H. pylori* in the pathogenesis of diabetes and its complications, but the results are inconsistent with each other. For example, some case-control studies have reported higher prevalence of *H. pylori* in patients with diabetes [14, 15]. Also, several cross-sectional studies have shown a significant statistical association between *H. pylori* and diabetes [3, 15]. However, some studies in this regard have shown that there is no significant association between diabetes and prevalence *H. pylori* infection [2, 16, 17].

Therefore, the association between *H. pylori* infection and the risk of diabetes is still controversies. Hence, this systematic review and meta-analysis study was designed to identify the possible association between *H. pylori* infection and the risk of diabetes.

## Methods

This systematic review and Meta-analysis was performed according to the Preferred Reporting Items for Systematic Reviews and Meta-analyses (PRISMA) and Strengthening the Reporting of Observationally Studies in Epidemiology (STROBE) guidelines for reviews of analytical observational studies (case-control) [18–20].

### Search terms and complex search syntax

**Fig. 1:**
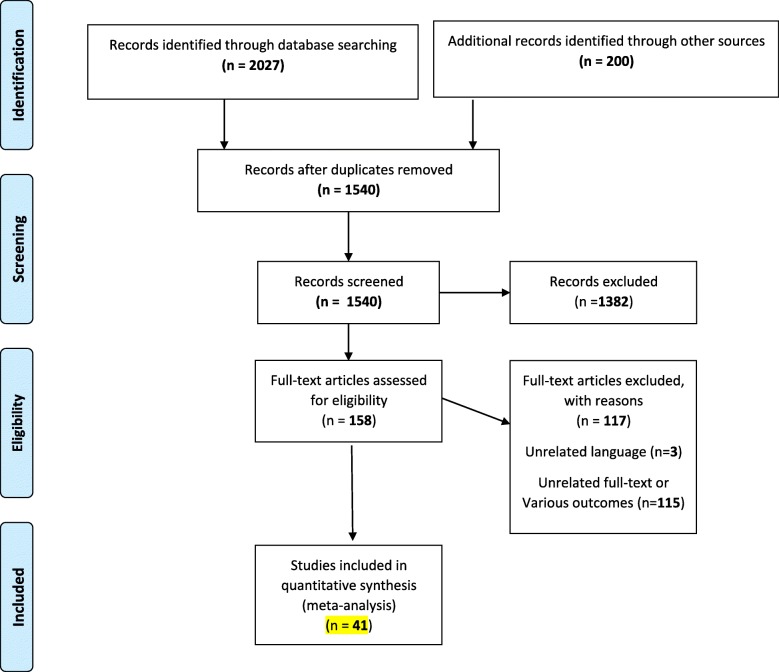
Flow Diagram of the Literature Search and Study Selection

All original published articles were searched from January 1990 to March 2019 without language limitations in international databases, including Medline (PubMed), Web of Sciences, Scopus, EMBASE, Cochrane, Ovid and CINHAL. The keywords were Diabetes, Diabetes Mellitus (type 1 and 2), Insulin Dependent, IDDM, NIDDM, Noninsulin Dependent, Insulin Sensitivity, Helicobacter pylori, Campylobacter pylori, and H Pylori. Two reviewers (YM and RR) abstracted data independently and reached consensus on all items. Inclusion and exclusion criteria were set by two researchers separately (YM and RR) (Fig. 1).

### Eligibility criteria

A published study had to meet the following inclusion criteria:

(1) case-control, nested case control studies, (2) human population, and (3) Helicobacter pylori infection was exposure variable. Case reports, reviews, animal studies, and cohort studies were removed from the tabulation. The authors resolved all disputes during the collection, compilation, and analysis of data.

### Data extraction

Two reviewers (YM and RR) independently extracted the data. A structured checklist was used for the extraction of information including; 1) name of first author, 2) date of publication, 3) country, 4) study subjects, 5) age of patients, 6) sample size, 7) type of diabetes, 8) mean of HbA1C, 9) duration of diabetes, 10) measurement of association, 11) controlled variables, 12) and method of bacteria detection. In addition, type of instruments (detection of *H. pylori*) was extracted from each study in case of reporting. A data extraction form was created based on our group discussion and piloted according to 10 different types of studies. Then, it was modified and used by the data extractor. All process from systematic search to final data extraction were followed independently by two research experts (Kappa statistic for agreement for quality assessment; 0.75). Any disagreement was assessed by both and if a consensus was not reached, a third author (LS) evaluate the study. The qualities of all studies were assessed by Modified Newcastle-Ottawa Scale for Case Control studies [21].

### Statistical analysis

Logarithm and standard error logarithm odds ratio (OR) were used for the meta-analysis. DerSimonian and Laird method was used to compute the pooled estimate of odds ratio (OR) with confidence interval (CI 95%) using random models [22]. Because the test for heterogeneity was statistically significant in some analyses, the random effects models were used to estimate OR. In this study, w Cochran’s Q test and I2 statistic were used to evaluate statistical heterogeneity between studies [23]. In addition, a meta-regression and subgroup analysis were performed to assess the source of heterogeneity between studies. Moreover, publication bias was assessed by funnel plot and Egger test [24, 25]. Statistical analysis was performed using STATA 14.0 (Stata Corp, College Station, TX, USA), and statistical significance was set at *p* < 0.05.

## Results

### Study characteristics

The first step of search in electronic databases yielded 2027 publications and 200 studies identified through other sources. In the final step, after removing the duplicates, reviewing by title, abstract and full text and considering the inclusion and exclusion criteria, 41 studies were selected for the meta-analysis of pooled association between *H. pylori* infection and the risk of diabetes (Fig. 1). Characteristics of included studies in this meta-analysis are reported in Table 1. The total sample size in the 41 studies that reported the association between *H. pylori* infection and the risk of diabetes in case and control was 4327 and 5232, respectively. Also, 11 studies reported association between *H. pylori* infection with DM. Other primary studies reported association between *H. pylori* infection with type 1 and 2 diabetes. A total of 41 studies were included in this meta-analysis, of which 20 were conduct in European, 12 were in Asian, 7 studies done in African and 2 in American (Table 2). Of the 41 studies, 18 showed statistically significant between *H. pylori* infection and the risk of diabetes.

**Table 1:** The main characteristics of Case – Control studies of the effect of H pylori on risk of diabetes

*Authors*	*Years*	*Country*	*Control subjects(selection methods)*	*Age*	*Sample size*	*Type of Diabetic* *(Mean HbA1C)* *(Duration of disease)*	*Measurement of association* *Odds Ratio* *(CI 95%)*	*Controlled variables*	*Bacteria detection*	*NOS* *Score*
Małecki, M. et al. [26]	1996	Poland	Non-diabetic subjects	17–80	139 (Control:100 & Case: 39)	DM (−) (8 Year)	0.33 (0.18, 0.59)	–	Histology or biopsy	6
Pocecco, M. et al. [27]	1997	Italy	Admitted for minor extra-abdominal surgery with no history of abdominal pain	16	379 [Control:310 & Case: 69]	DM (−) (−)	3.13 (2.08, 4.70)	Age, sex, education and economic	Rapid urease test	6
Gentile, S [28].	1998	Italy	Non-diabetic subjects	52	328 (Control:164 & Case: 164)	T2DM (8.3 ± 1.4)	1.77 (1.35, 2.31)	Age, sex and body weight	Histology or biopsy	7
De Luis, DA [29]	1998	Spain	The control subjects were healthy volunteers, with similar age and sex-distribution that the diabetic patients	25	180 Control: 100 & Case: 80	T1DM (−) (3.1 Year)	1.36 (0.98, 1.87)	Age and sex	Anti-*H. pylori* antibody	6
Gasbarrini, A.et al. [30]	1998	Italy	Healthy subjects	35	166 [Control: 50 & Case: 116]	DM (−) (19 year)	1.04 (0.85, 1.28)	Age and sex	13C or 14C urea breath test	6
Salardi, S.et al. [31]	1999	Italy	Children with minor endocrine disorders.	12	339 [Control: 236 & Case: 103]	T1DM (−) (−)	1.47 (0.99, 2.18)	Age	Anti H. pylori antibody	7
Arslan, D. et al. [32]	2000	Turkey	Non-diabetic subjects	12	130 (Control: 42 & Case: 88)	T1DM (11.08 ± 3.17) (3.85 Year)	1.38 (1.08, 1.75)	–	Anti-H. pylori antibody	6
Dore, MP. et al. [33]	2000	Italy	Blood donors from the same geographic area	12–75	891 [Control: 506 & Case: 385]	DM (greater than 1 year)	1.16 (1.00, 1.35)	age and socioeconomic status	Anti-H. pylori antibody	8
Senturk, O. et al. [34]	2001	Turkey	Nondiabetic patients undergoing upper diagnostic endoscopies	54.1	140 [Control: 73 & Case: 67]	T2DM (6.42 ± 0.97) (4.5 year)	1.39 (0.78, 2.48)	Age and socioeconomic	Histology or biopsy	7
Ravera, M.et al. [35]	2001	Uganda	Dyspeptic patients without diabetic	–	132 [Control110: & Case: 22]	DM (−) (−)	1.22 (0.33, 4.49)	–	Histology or biopsy	6
Ko, G. T.et al. [36]	2001	Chine	With upper GI symptoms in whom	49.9	118 [Control55: & Case: 63]	T2DM (8.25 ± 2.22) (6.2 year)	0.90 (0.64, 1.26)	Age and sex	Rapid urease test	6
Marrollo M.et al. [37]	2001	Italy	Non diabetic dyspeptic patients	63	191 [Control: 117& Case: 74]	DM (−) (−)	1.54 (1.05, 2.27)	Age and sex	Rapid urease test and Histology or biopsy	6
Quatrini, M.et al. [38]	2001	Italy	Dyspepsia patients	58	142 [Control: 71 & Case: 71]	DM (−) (−)	1.63 (1.12, 2.38)	Age and sex	13C or 14C urea breath test	7
Cenerelli, S. et al. [39]	2002	Italy	Control subjects were first selected on the basis of the admission criteria of the senieur protocol.	55	73 Control: 43 & Case: 30)	T2DM (6.1 ± 1.8) (3.1 Year)	1.04 (0.60, 1.80)	–	13C or 14C urea breath test	7
Maule, S.et al. [40]	2002	Italy	Individuals without diabetes	46–75	62 [Control:31 & Case: 31]	T2DM (7.1 ± 1.4) (−)	1.65 (0.92, 2.97)	Age	13C or 14C urea breath test	8
Candelli, M. et al. [41]	2003	Italy	The control Group was selected normal healthy adolescent	17	268 Control: 147 & Case: 121)	T1DM (8.2 ± 1.4) (6.7 Year)	0.97 (0.72, 1.30)	Sex, age and social class	Rapid urease test, Histology or biopsy	7
Gulcelik, N. E.et al. [4]	2005	Turkey	Dyspeptic non diabetic subjects	51.9	149 [Control: 71 & Case: 78]	T2DM (8.2 ± 1.4)	1.92 (1.29, 2.86)	Age and BMI	Histology or biopsy	7
Jaber, S. M.et al. [42]	2006	Saudi Arabia	Healthy children	> 10	604 [Control:543 & Case: 61]	T1DM (−) (−)	1.60 (0.98, 2.63)	–	Anti H. pylori antibody	6
Bener, A. et al. [43]	2007	Qatar	Non-diabetic subjects	48	420 (Control:210 & Case: 210)	T2DM (6.9 ± 1.4) (−)	5.03 (3.90, 6.47)	Age and sex	Anti-H. pylori antibody	7
Demir, M. et al. [44]	2008	Turkey	The control Subjects were selected in the gastroenterology clinics	52	283 Control: 142 & Case: 141	T2DM (−) (6 year)	1.07 (0.84, 1.36)	Age and sex	Rapid urease test and Histology or biopsy	7
Ariizumi, K. et al. [45]	2008	Japan	non-diabetic subjects without upper GI tract disorders	62	134 [Control: 67 & Case: 67]	DM (−) (15.1 year)	0.74 (0.53, 1.03)	age and sex-matched	Anti H. pylori antibody, Rapid urease test, Histology or biopsy	8
Hamed, S. A.et al. [46]	2008	Egypt	Subjects with neither history nor clinical evidence of gastrointestinal problems; vascular, inflammatory, or neurologic diseases.	47.6	140 [Control:60 & Case: 80]	DM (−) (9.2 year)	1.29 (0.83, 2.01)	Age and sex	Anti H. pylori antibody	8
Cabral, V. L. R. et al. [47]	2009	Brazil	The control Group was selected normal healthy adolescent	17	45 Control: 30 & Case: 15)	T1DM (−) (−)	0.52 (0.21, 1.29)	–	Histology or biopsy	7
Lazaraki, G. et al. [48]	2009	Greece	non-smoking, non-diabetic with of dyspepsia	65	79 [Control: 30 & Case: 49]	T2DM (−) (3 year)	0.99 (0.70, 1.40)	Age, sex, H. pylori-infection, degree of gastritis	Rapid urease test and Histology or biopsy	7
Krause, I. et al. [49]	2009	Colombia	Individuals had no clinical diabetes, nor islet cell autoantibodies	16.0	180 [Control: 123: & Case: 57]	T1DM (−) (8.8 year)	0.44 (0.29, 0.66)	–	Anti-H. pylori antibody	6
Devrajani, BR. et al. [15]	2010	Pakistan	Non diabetic individuals with positive or negative Helicobacter pylori infection	53	148 [Control: 74 & Case: 74]	T2DM (−) (5 years)	1.64 (1.11, 2.43)	–	Stool antigen test	7
Ibrahim, A. et al. [50]	2010	Egypt	Dyspeptic non diabetic subjects	45	200 [Control: 102 & Case: 98]	T2DM (8.57 ± 0.79) (−)	0.94 (0.71, 1.25)	–	Rapid urease test, Histology or biopsy	7
El-Eshmawy, M. M. et al. [51]	2011	Egypt	Non-diabetic subjects	20	242 (Control:80 & Case: 162)	T1DM (8.2 ± 1.75) (7.29 Year)	1.63 (1.25, 2.11)	Age, sex, geographic area and socioeconomic status	Anti-H. pylori antibody	7
De Block, C. E. M. et al. [52]	2012	Belgium	One-hundred sex- and age-matched controls were tested for H. pylori serology.	40	329 Control: 100 & Case: 229)	T1DM (7.8 ± 1.0) (18 Year)	0.86 (0.74, 1.02)	Age and sex	Anti-H. pylori antibody & Rapid urease test and Histology or biopsy	7
Candelli, M. et al. [53]	2012	Italy	Healthy children	19.8	174 [Control: 99 & Case: 75]	T1DM (8.8 ± 0.80) (−)	1.96 (1.40, 2.75)	Age, sex and socio-economic	13C or 14C urea breath test	6
Jafarzadeh, A.et al. [54]	2012	Iran	Healthy individuals	42.86	200 [Control: 100 & Case: 100]	T2DM (−) (−)	1.03 (0.74, 1.42)	Age	Anti H. pylori IgG	6
Keramat, F. et al. [55]	2013	Iran	Non-diabetic subjects	51	158 (Control: 79 & Case: 79)	DM (8.96 ± 1.82) (2.78 Year)	1.29 (0.89, 1.88)	Age and sex	Anti-H. pylori antibody & Rapid urease test and Histology or biopsy	8
Zekry, O. A. et al. [56]	2013	Egypt	Healthy children and adolescents	12.53	120 [Control: 60 & Case: 60]	T1DM (7.75 ± 1.67) (9.25 year)	1.69 (1.21, 2.35)	Age and sex	Anti-H. pylori antibody	8
Chobot, A. et al. [57]	2014	Poland	This group was enrolled from a large cohort of children	13.4	447 [Control: 298 & Case: 149]	T1DM (7.69 ± 1.63) (4.6 year)	0.74 (0.48, 1.15)	Age- and sex	13C or 14C urea breath test	8
Fayed, SB. et al. [58]	2014	Egypt	healthy normal volunteers	12.2	106 [Control:53 & Case: 53]	T1DM (9.6 ± 1.6) (12.2 year)	1.80 (1.14, 2.84)	Age and sex	Anti H. pylori antibodies	7
Zhou, F. et al. [17]	2015	China	Non-diabetic subjects with dyspepsia symptoms	45	253 (Control:65 & Case: 188)	T2DM (8.2 ± 1.9)	1.15 (0.99, 1.33)	Age and sex	Anti-H. pylori antibody & Rapid urease test	9
Bajaj, S. et al. [3]	2015	India	The control group comprised of age, sex, socioeconomic status, and education matched normal healthy volunteers	> 18	140 Control: 60 & Case: 80)	T2DM (8.2 ± 1.2) (4.2 Year)	1.53 (1.04, 2.24)	Age, sex, socioeconomic status, and education	Anti-H. pylori antibody & Rapid urease test and Histology or biopsy	8
Bazmamoun, H. et al. [59]	2016	Iran	Non-diabetic subjects	10	160 (Control: 80 & Case: 80)	T1DM (8.00 ± 0.65) (2.72 Year)	1.50 (1.09, 2.07)	Age, sex, socioeconomic status	Anti-H. pylori antibody	8
Osman, S. M.et al. [60]	2016	Sudan	Healthy children	1–18	180 [Control: 90 & Case: 90]	T1DM (−) (6 month)	0.97 (0.71, 1.33)	age and sex	Anti-H. pylori antibody	8
Alzahrani, S. et al. [61]	2017	Saudi Arabia	Non-diabetic subjects	49	842 (Control:421 & Case: 421)	DM (6.1 ± 0.6)	1.01 (0.88, 1.16)	Age, sex, race, DPP intervention, length of follow-up time, body mass index, alcohol consumption, physical activity and smoking	Anti-H. pylori antibody & Rapid urease test	9
Vaishnav, B. et al. [62]	2018	India	Non diabetic with dyspepsia	56	287 [Control: 140 & Case: 147]	T2DM (8.4 ± 1.0) (7.59 year)	1.89 (1.51, 2.36)	–	Rapid urease test	8

**Table 2:** Summary odds Ratio (OR) Estimates [95% confidence intervals (CIs)] for Case–Control studies Conducted on the Association Between Helicobacter pylori and Risk of diabetes by Type of diabetes, Continent, Mean of HbA1C, Duration of Diabetes, Method of detection bacteria, NOS score and Age

Subgroup	Number of studies	Summery Odds Ratio (95% CI)	Between studies			Between subgroups	
			I^2^	*P*_heterogeneity_	Q	Q	*P*_heterogeneity_
Type of diabetes							
Diabetes Mellitus	11	1.17 (0.94–1.45)	82.5%	0.0001	1.43		
Type 1 Diabetes	15	1.19 (0.98–1.45)	81.6%	0.0001	1.75	3.59	0.0001
Type 2 Diabetes	15	1.43 (1.11–1.85)	90.0%	0.0001	2.72		
Continent							
Asian	12	1.41 (1.05–1.88)	93.2%	0.0001	2.29		
American	2	0.45 (0.31–0.66)	0.0%	0.728	4.12	3.59	0.001
African	7	1.32 (1.05–1.66)	61.0%	0.018	2.40		
European	20	1.26 (1.08–1.47)	80.3%	0.0001	2.94		
Mean of HbA1C^a^							
6–8	9	1.40 (0.92–2.13)	95.0%	0.001	1.55		
8 <	13	1.41 (1.20–1.64)	73.7%	0.001	4.33	3.59	0.0001
Duration of Diabetes^a^							
0–3 Y	7	1.18 (1.06–1.31)	0.0%	0.450	3.07		
4–7 Y	10	1.15 (0.95–1.38)	69.1%	0.001	1.45	3.59	0.001
8 < Y	9	1.09 (0.79–1.51)	91.0%	0.0001	0.55		
Method of detection bacteria							
Invasive Tests	18	1.07 (0.93–1.23)	73.2%	0.0001	1.00		
Non- Invasive Test	23	1.45 (1.19–1.76)	88.5%	0.0001	3.70	3.59	0.0001
Age^a^							
10–30 Y	15	1.30 (1.05–1.62)	81.9%	0.0001	2.37		
30–60 Y	18	1.34 (1.09–1.65)	91.3%	0.0001	2.77	3.59	0.0001
60 < Y	3	1.03 (0.68–1.57)	76.4%	0.014	0.15		
NOS Score							
6	11	1.14 (0.85–1.53)	87.7%	0.0001	0.86		
7	16	1.42 (1.10–1.82)	89.0%	0.0001	2.72		
8	12	1.24 (1.00–1.53)	81.6%	0.0001	1.96	3.59	0.0001
9	2	1.08 (0.95–1.22)	35.0%	0.216	1.14		

Largely diabetes mellitus

All statistical tests were 2-sided

^a^other studies not reported HbA1C, duration of diabetes,

**Fig. 2:**
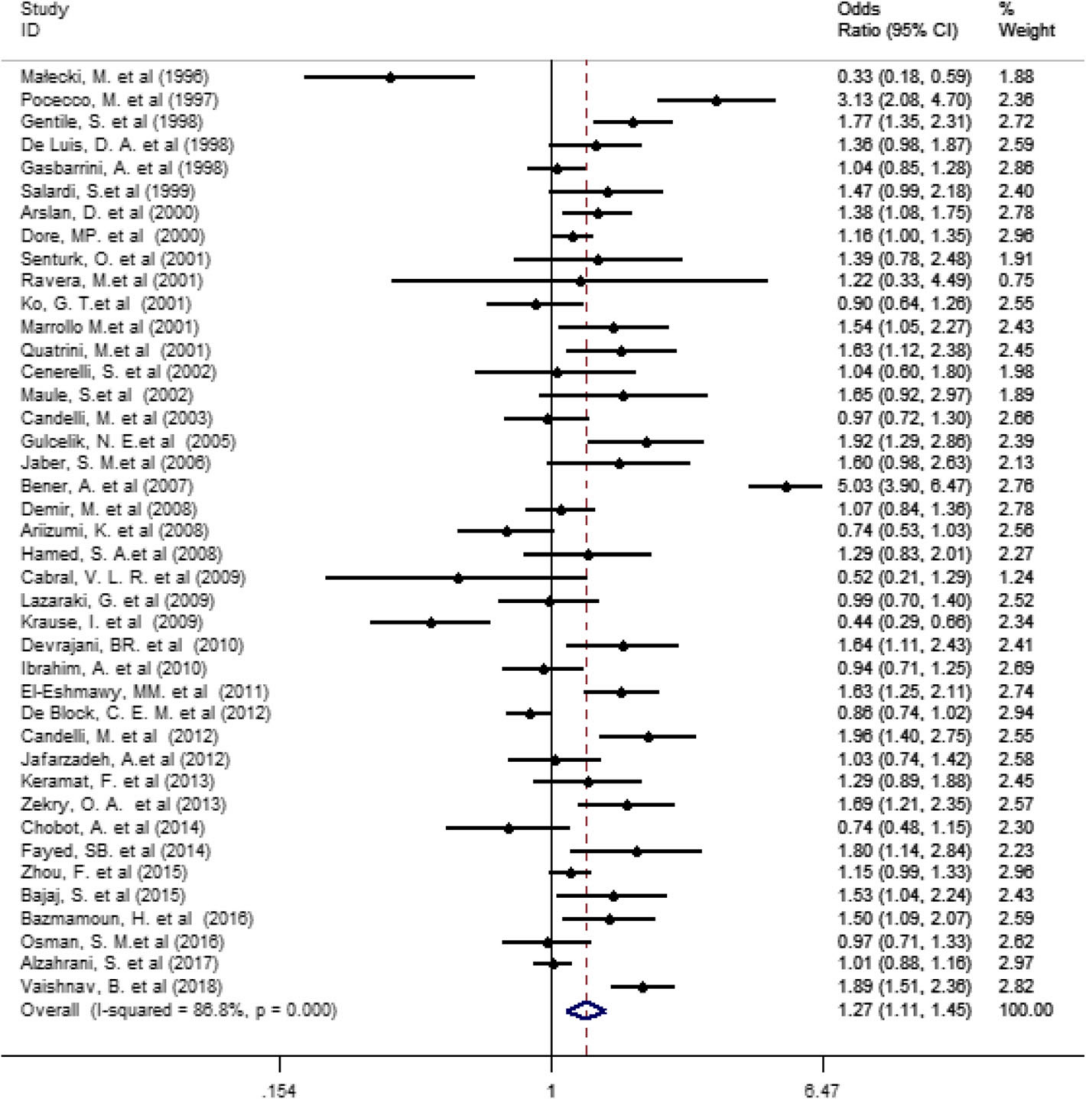
Association between Helicobacter pylori and Risk of diabetes (DM, T2DM and T1DM)

**Fig. 3:**
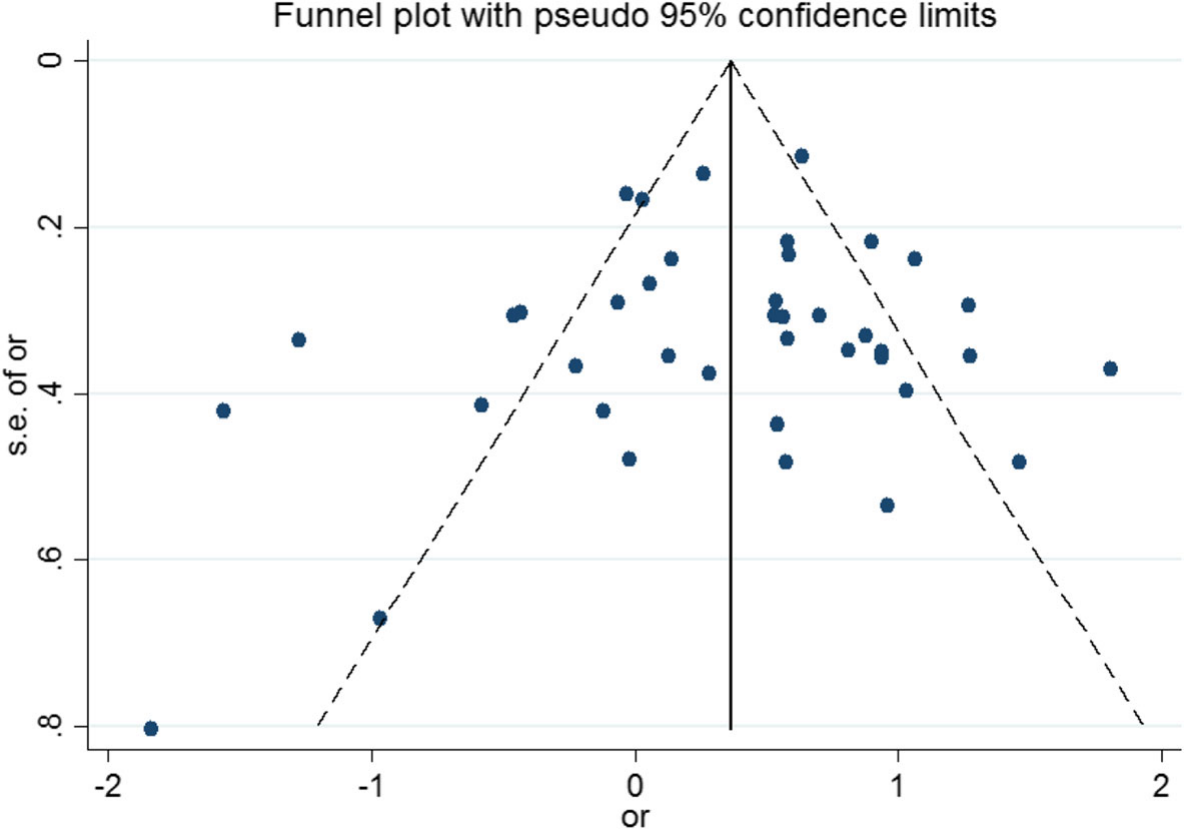
Funnel plot of association between Helicobacter pylori and Risk of diabete**s**

The pooled estimate of the association between *H. pylori* infection with diabetes mellitus was 1.27 (95% CI 1.11 to 1.45, *P* = 0.0001, I^2^ = 86.6%) (Fig. 2), but since the CI of test (Egger’s test) included zero, no significant bias occurred in the publication of the results (Egger’s test = 1.579, *P* = 0.073, 95% CI − 0.154 to 3.312) (Fig. 3).

### Subgroup analysis

Based on the random effect model, the effect of *H. pylori* infection on diabetes mellitus, type 1 and type 2 diabetes was 1.17 (95% CI 0.94 to 1.45), 1.19 (95% CI 0.98 to 1.45), and 1.43 (95% CI 1.11 to 1.85) respectively. Effect size of *H. pylori* infection on type 2 diabetes was higher than type 1 and diabetes mellitus (Table 2). Some of the primary studies did not mention that the study population was type 1 or type 2, therefore we decided to include these types of studies as diabetes mellitus group in subgroup analysis.

Subgroup analysis by the geographical regions showed in Asian population risk of the effect of *H. pylori* infection on diabetes was higher than other population. In addition, the relationship between *H. pylori* and the risk of diabetes according to age showed that risk in individual with 30 to 60 years was 1.34 (95% CI 1.09, 1.65), in 10 to 30 years, and upper 60 years was 1.34 (95% CI 1.05, 1.62) and 1.03 (95% CI 0.93, 3.23), respectively (Table 2).

The effect of *H. pylori* infection on diabetes mellitus by non- invasive testes detected was higher than invasive tests methods, (Table 2).

## Discussion

Our systematic review and meta-analysis suggest that there is a positive association between *H. pylori* infection and diabetes. The results showed a significant statistical association between *H. pylori* infection and the risk of diabetes (overall OR: 1.27; 95% CI: 1.11–1.45). The results of subgroup analysis by type of diabetes revealed a significant association between *H. pylori* infection and the risk of type 2 diabetes (OR: 1.43; 95% CI: 1.11–1.85). However, this meta-analysis showed a positive relationship between *H. pylori* and the risk of type 1 diabetes (OR: 1.19; 95% CI: 0.98–1.45) and diabetes mellitus (OR: 1.17; 95% CI: 0.94–1.45) but statistically were not significant. Subgroup analysis by the geographical regions showed a significant direct relationship between *H. pylori* and the risk of diabetes in Asian, Europe and Africa but in the American population this association was negative. In addition, in subgroup analysis, the relationship between *H. pylori* and the risk of diabetes was different based to age, level of HbA1C, duration of diabetes and methods for *H. pylori* detection. This suggests that these factors could be an important source of heterogeneity in the studies included in the meta-analysis.

Our meta-analysis suggests that *H. pylori* infection may increase the risk of diabetes by up to 27%. These findings are consistent with the results of several meta-analysis studies that have been done in this field. According to our knowledge, three other meta-analysis studies have been conducted regarding the association between *H. pylori* and diabetes. The first study which has been conducted by Jun-Zhen Li on 79 studies with 57,397 individuals from January 1996 to January 2016, showed that the prevalence of *H. pylori* infection in diabetes mellitus patients was significantly higher than non-diabetic (OR: 1.69; 95% CI: 1.47–1.95), however, this difference was only significant for type 2 diabetes (OR: 2.05; 95% CI: 1.67–2.52) [2]. The second study that was carried out by FENG WANG on 39 eligible studies for meta-analysis form 1997 and 2012, revealed that the *H. pylori* infection also associated with increased risks of type 1 (OR: 1.99, 95% CI 1.52–2.60) and type 2 diabetes (OR: 2.15, 95% CI 1.81–2.55) [63]. In the third meta-analysis study by Zhou et al. on 41 articles and 14,080 participants, the results reveled a significant association between *H. pylori* infection and increased risks of diabetes (OR: 1.33; 95% CI: 1.08–1.64) [64]. However, some studies did support significant association between *H. pylori* infection and the risk of diabetes [16, 65].

Several mechanisms have been proposed for the relationship between *H. pylori* infection and risk of diabetes. Inflammatory cytokine may lead to induce phosphorylation of serine residues on the insulin receptor substrate and subsequently this phenomenon may impair the interaction between the substrate and the insulin receptors due to impaired insulin function [7, 66]. Also, Lipopolysaccharides from gram-negative bacteria such as *H. pylori* may activate Toll-like receptors and subsequently insulin resistance occurs [67]. All of these events can lead to reduced blood sugar control and consequently diabetes mellitus. In addition, the presence of bacterial infections can lead to microvascular failure and eventually incidence of atherosclerosis [68].

In subgroup analysis of geographical regions by the type of continent, we explored a significant direct relationship between *H. pylori* and the risk of diabetes in Asian, Europe and Africa but not in the American, however there was still high heterogeneity within these subgroups, therefore the interpretation of this negative result would be difficult. This was consistent with study of Jun-Zhen Li et al. that have shown *H. pylori* infection is significantly higher in patients with diabetes residing in Asia and Europe than in Africa and the American [69]. Also, Wang F et al. reported *H. pylori* can increase the risk of diabetes in European, Middle East and South Asia [63]. But, study carried out by Zhou et al. found *H. pylori* infection is significantly higher in patients with diabetes residing in only Asia [64]. This difference in various continents may be due to differences in sample size, different diagnostic methods and different medical care conditions. However, to determine the precise effect of geographical location on the association between *H. pylori* and diabetes risk, it is suggested to carry out further studies to look at the role of genetic and environmental factors particularly in migrant populations.

Also, in subgroup analysis, we found a significant direct relationship between *H. pylori* and the risk of diabetes in mean of HbA1C > 8. This result was in line with the results of other studies in this field. For example, the study by Ming-Chia Hsieh et al. revealed that there is a significant and direct statistical relationship between prevalence of *H. pylori* and serum HbA1c levels but not fasting glucose levels after adjusting for sex, age, BMI and family history of diabetes mellitus, so that the positive *H. pylori* group had significantly higher serum HbA1c levels compared to the negative *H. pylori* group (5.78% vs. 5.69%, *p* = 0.007) [70]. Another study in China revealed individuals with *H. pylori* infection had a higher level HbA1C than those who did not [71]. Considering the HbA1c is a valid and reliable indicator for estimating average blood sugar in long-term, it seems to be more valid to evaluate the effect of chronic *H. pylori* infection on blood glucose regulation, because fasting glucose levels are subject to daily changes such as diet and physical activity which these fluctuations may impair any association between *H. pylori* infection and glucose regulation [72–74]. In addition, in subgroup analysis relationship between *H. pylori* and the risk of diabetes was different by age. This finding was consistent with results of other reports, because the different studies have shown that the prevalence of *H. pylori* infection varies with age [75].

Finally, association between *H. pylori* and the risk of diabetes was different by methods for *H. pylori* detection in subgroup analysis. This suggests that this factor could be an important source of heterogeneity in the studies included in the meta-analysis, because different methods of detection for *H. pylori* had different accuracy and precision in such cases the serological tests of anti- *H. pylori* IgG or/and IgA antibody in serum may be reported with different degree of false positives [76, 77]. As a result, association *H. pylori* and the risk of diabetes may be different according to the method of diagnosis of infection.

### Strengths and limitations

This study similar to other studies has some limitations and strengths.

One of the issue which make distinguish this meta-analysis with the previous ones is dealing with heterogeneity through a subgroups analysis based on type of diabetes, geographical regions, age, and level of HbA1c, duration of diabetes and detection methods of *H. pylori*. Another strength point of our meta-analysis is considering as much as reported and published studies in comparison with other systematic reviews (41 studies) that we were unable to investigate the exact effect of the publication bias.

Also, this study has some limitations. Firstly, missing potential studies e.g. limiting full- text review to English language articles may be lead to some degree of selection bias. Secondly, all studies included in meta-analysis were case-control, hence, the design and implementation of cohort studies are essential for detailed assessment of the association between *H. pylori* infection and diabetes. Thirdly, personal judgments may be effect on search of articles, data extraction and assessment of included articles in meta-analysis.

## Conclusion

According to this systematic review & meta-analysis, it can be concluded that *H. pylori* infection could be a potential risk factor for diabetes particularly type 2 diabetes, however further prospective studies are necessary to show the direction of this association.

## Data Availability

Input data for the analyses are available from the corresponding author on request.

## Abbreviations

CI: Confidence Interval
OR: Odds Ratio
IDDM: Insulin-Dependent Diabetes Mellitus
NIDDM: Non-Insulin-Dependent Diabetes Mellitus
CINAHL: Cumulative Index to Nursing and Allied Health Literature
EMBASE: Excerpta Medica dataBASE
STROBE: Strengthening the Reporting of Observationally Studies in Epidemiology
PRISMA: Preferred reporting items for systematic reviews and meta-analyses
*H. pylori*: Helicobacter Pylori
